# MEG activity of the dorsolateral prefrontal cortex during optic flow stimulations detects mild cognitive impairment due to Alzheimer’s disease

**DOI:** 10.1371/journal.pone.0259677

**Published:** 2021-11-05

**Authors:** Moeko Noguchi-Shinohara, Masato Koike, Hirofumi Morise, Kiwamu Kudo, Shoko Tsuchimine, Junji Komatsu, Chiemi Abe, Sachiko Kitagawa, Yoshihisa Ikeda, Masahito Yamada

**Affiliations:** 1 Department of Neurology and Neurobiology of Aging, Kanazawa University Graduate School of Medical Sciences, Kanazawa University, Kanazawa, Japan; 2 Department of Preemptive Medicine of Dementia, Kanazawa University Graduate School of Medical Sciences, Kanazawa University, Kanazawa, Japan; 3 Medical Imaging Business Center, Ricoh Company, Ltd., Tokyo, Japan; 4 Kudanzaka Hospital, Tokyo, Japan; Niigata University, JAPAN

## Abstract

Dorsal stream, which has a neuronal connection with dorsolateral prefrontal cortex (DLPFC), is known to be responsible for detection of motion including optic flow perception. Using magnetoencephalography (MEG), this study aimed to examine neural responses to optic flow stimuli with looming motion in the DLPFC in patients with mild cognitive impairment due to Alzheimer’s disease (AD-MCI) compared with cognitively unimpaired participants (CU). We analyzed the neural responses by evaluating maximum source-localized power for the AD-MCI group (n = 11) and CU (n = 20), focusing on six regions of interest (ROIs) that form the DLPFC: right and left dorsal Brodmann area 9/46 (A9/46d), Brodmann area 46 (A46) and ventral Brodmann area 9/46 (A9/46v). We found significant differences in the maximum power between the groups in the left A46 and A9/46v. Moreover, in the left A9/46v, the maximum power significantly correlated with the Wechsler Memory Scale-Revised general memory score and delayed recall score. The maximum power in the left A9/46v also revealed high performance in AD-MCI versus CU classification with the area under the ROC curve of 0.90. This study demonstrated that MEG during the optic flow task can be useful in discriminating AD-MCI from CU.

## Introduction

Patients with Alzheimer’s disease (AD) commonly have visuospatial problems [1], which can impair the activities of daily living [2]. Motion perception such as looming is important for adapting behavior in an environment, hence it is essential to raise awareness about these issues early to maintain safe living.

**Fig 1:**
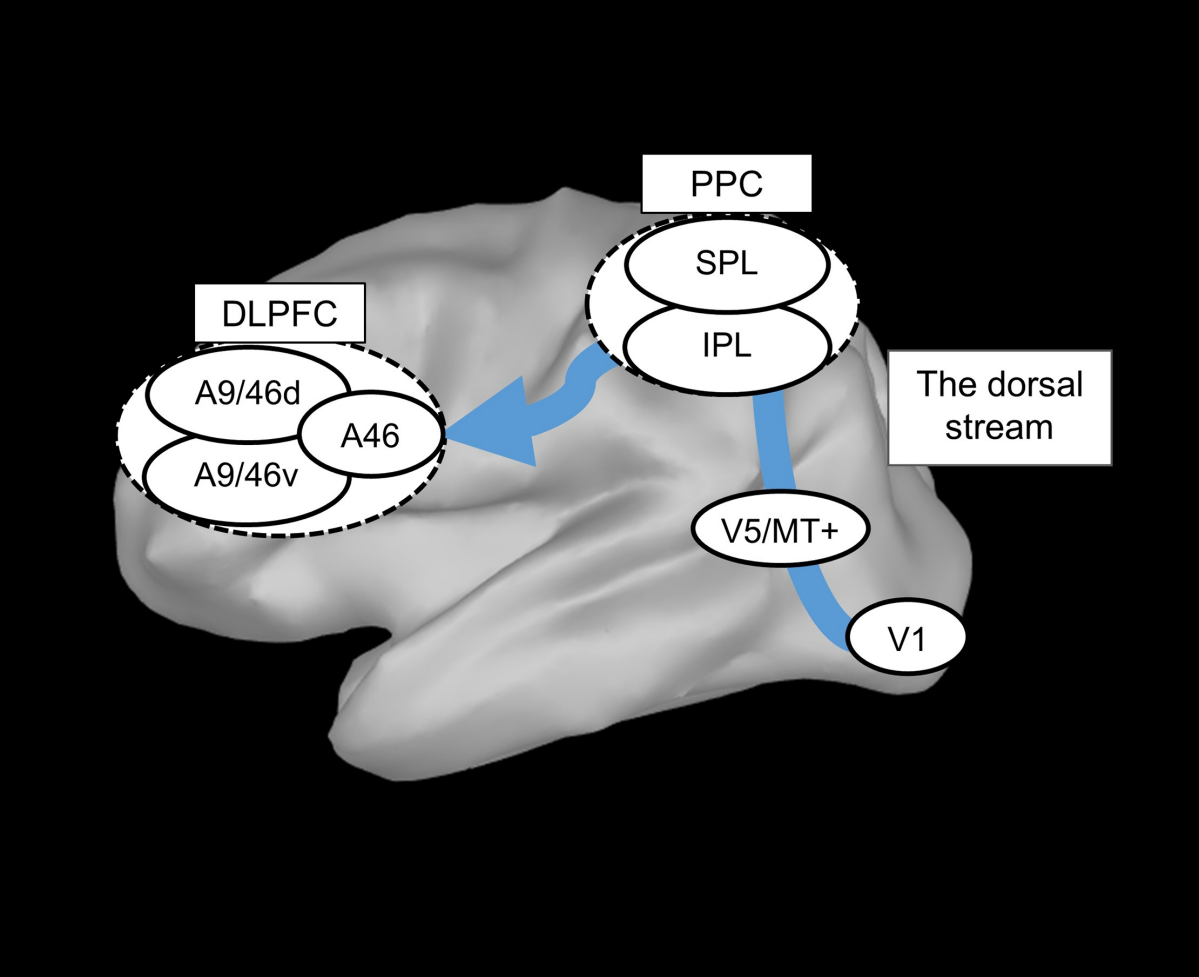
Flow of neural signals during optic flow perception. The dorsal stream, which includes visual cortex V1, V5/MT+ and PPC (SPL and IPL), is responsible for detecting the motion of optic flow perception. PPC has a connection with DLPFC, which includes A9/46d, A46 and A9/46v. Abbreviations: A9/46d, Dorsal Broadmann area 9/46; A9/46v, Ventral Broadmann area 9/46; A46, Broadmann area 46; DLPFC, dorsolateral prefrontal cortex; IPL, inferior parietal lobule; PPC, posterior parietal cortex; SPL, superior parietal lobule; V1, visual cortex1; V5/MT+, visual cortex5/middle temporal.

It has been reported that a considerable proportion of patients with mild cognitive impairment (MCI) and AD dementia have impaired perception of optic flow to simulate looming [3, 4]. The dorsal stream was reported to be responsible for the detection of motion, including optic flow perception (Fig 1) [5].

Primate studies, however, have reported that optic flow stimuli with looming motion activated a large cortical network in frontal, parietal and occipital cortex in areas involved in the analysis of motion, identity and attention [6]. This indicated that optic flow perception might be associated with a broad cortical network including the dorsal stream in humans. The dorsal stream is composed of visual cortex (V) 1, V5/middle temporal (V5/MT+) and the posterior parietal cortex (PPC) which consists of superior parietal lobule (SPL) and inferior parietal lobule (IPL) [7], and it has a connection with the dorsolateral prefrontal cortex (DLPFC) through the PPC [8, 9]. The DLPFC has been considered a source of visuospatial attention signals providing selection bias in the early visual areas in favor of the attended features [10]. Considering the connection between the dorsal stream and DLPFC, the optic-flow task as motion detection may induce activity in the DLPFC as well as the dorsal stream. Actually, several functional magnetic resonance imaging (fMRI) studies have indicated the activation in the DLPFC activation during optic flow stimulation [11, 12].

A functional near-infrared spectroscopy (fNIRS) study reported that older adults showed significantly increased activation in the DLPFC compared with younger adults during optic flow stimuli [13]. The visuospatial attention signals are believed to be cholinergically mediated [14], and they are vulnerable to the cholinergic decline associated with aging, as well as to the cholinergic disruption that characterizes neurodegenerative conditions, such as MCI and AD [15]. These cholinergic vulnerability of visuospatial attention signals because of aging and/or neurodegeneration may alter the brain activity in the DLPFC.

The aim of the present study was to obtain a better understanding of the mechanisms of cortical processing of optic flow stimuli with looming motion information in patients with MCI due to AD (AD-MCI). The number of patients with AD has markedly increased as the population has aged, and the importance of detection of AD-MCI, which is prodromal stage of AD dementia, has been enhanced. We were especially interested in DLPFC, because it has been implicated not only in working memory, but also in multisensory processing, sustained attention, and decision making [8, 16, 17]. In addition, alterations in working memory and decision making have been reported as one of the earliest signs of AD-MCI [18]. We therefore hypothesized that AD-MCI might be associated with the alteration in the brain functions of the DLPFC during optic flow with the progress of cognitive decline. Here we demonstrated that magnetoencephalography (MEG) analyses of the brain activity in the DLPFC during optic flow would be clinically useful to distinguish AD-MCI from cognitively normal older adults.

## Materials and methods

### Study population

According to the diagnostic guidelines for AD from the National Institute on Aging-Alzheimer’s Association [19, 20], we recruited patients diagnosed as having MCI with an intermediate or high likelihood of AD, as assessed with biomarkers; biomarkers of amyloid β (Aβ) included cerebrospinal fluid (CSF) Aβ_42_ and amyloid positron emission tomography (PET) imaging, and biomarkers of tau and neurodegeneration/ neuronal injury refer to CSF phosphorylated tau, medial temporal lobe atrophy (MTA) on MRI, and temporoparietal/ precuneus hypometabolism on fluorodeoxyglucose-PET. Regarding CSF markers, we set the cutoff value of 490 pg/mL and 49 pg/mL for CSF Aβ_42_ and CSF phosphorylated tau, respectively, in our laboratory as previously described in a published work from our laboratory [21]. For evaluation of amyloid PET, we considered cortical amyloid deposition such as unilateral binding in one or more cortical brain region to be positive for AD pathology [22]. For evaluation of FDG-PET, we used the following visual criteria for assessment of neurodegeneration: reduction in the metabolism in the temporoparietal lobe, precuneus and/or posterior cingulate gyrus and relative sparing of the metabolism in the sensorimotor cortex, occipital cortex and cerebellum [21]. For evaluation of atrophy of the medial temporal lobe, we used the visual rating scale of MTA [23]. MTA was rated on the coronal T1-weighted images using a 5-point visual rating scale, ranging from 0 (no atrophy) to 4 (severe atrophy) based on the height of the hippocampal formation and the surrounding CSF space [23]. If the MTA was ≥ 2, we considered it as neurodegeneration [23]. All patients were examined by neurologists, and patients receiving medications that act upon the central nervous system (i.e., cholinesterase inhibitors, N-Methyl-d-aspartate receptor antagonists, antipsychotics, anticholinergics, or antidepressants) were excluded from the study. The brain MRI was performed to eliminate any other potential medical conditions. Cognitively unimpaired participants (CU) were recruited from participants in the Nakajima study. This is a population-based longitudinal cohort study that investigated cognitive decline in residents aged ≥ 60 years in Nakajima, Ishikawa Prefecture, Japan [24]. The CU group had no history of psychiatric or neurological diseases and were receiving no medications that could act on their central nervous system. All subjects were assessed to be cognitively unimpaired. The cognitive profiles were evaluated using the Mini-Mental State Examination (MMSE) [25] and Wechsler Memory Scale Revised (WMS-R) [26]. Regarding the visuospatial problems, we assessed the difficulty in drawing the intersecting pentagon copying from the MMSE. All participants were right-handed except for three patients with AD-MCI (left-handed).

### Standard protocol approvals and participant consent

This study was conducted according to the guidelines of the Declaration of Helsinki and all procedures involving human subjects were approved by the Kanazawa University Medical Ethics Review Board (approval number 2918). Written informed consent was obtained from all subjects.

### Optic flow stimuli

**Fig 2:**
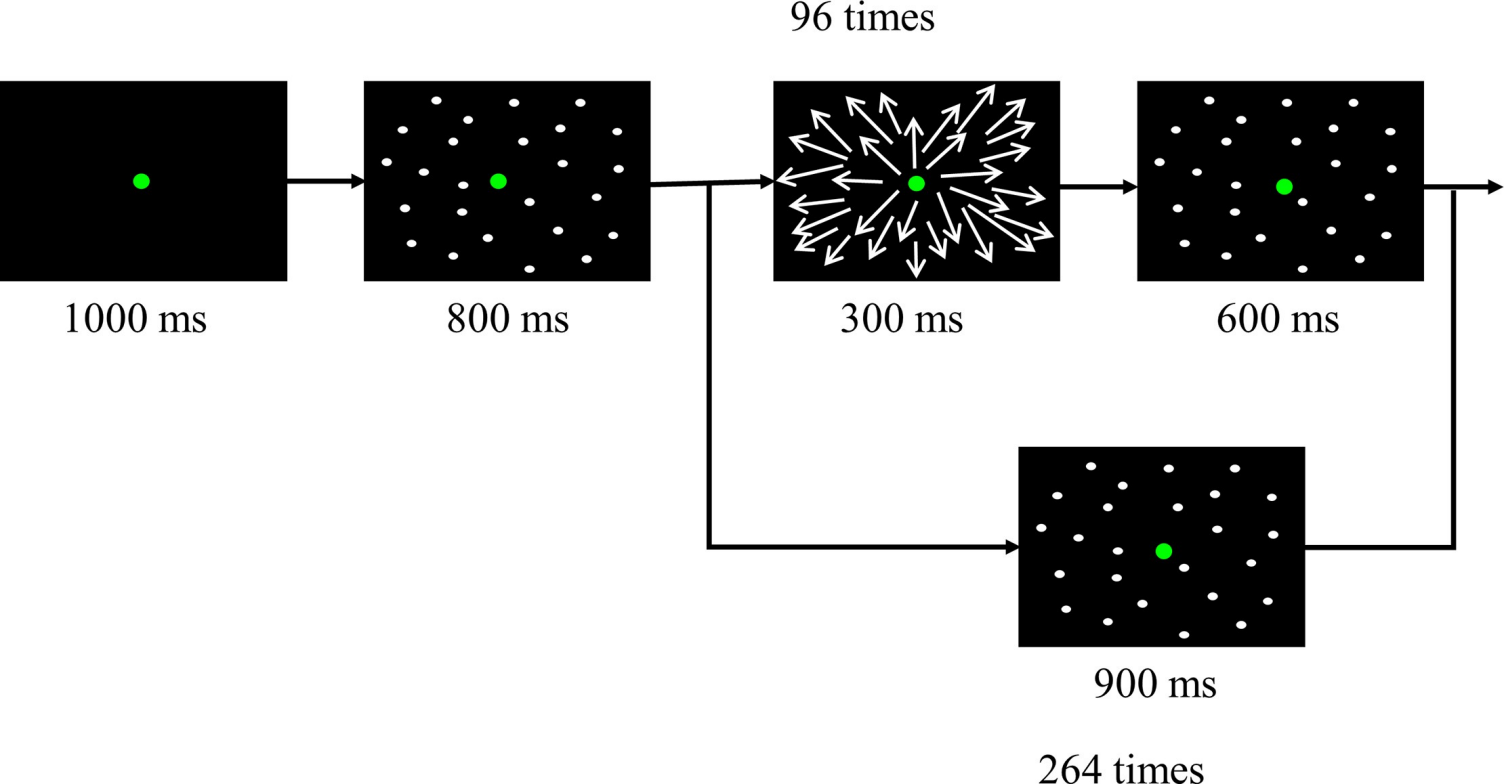
Optic flow stimuli. The optic flow stimuli started with a centered fixation spot for 1000 ms. It was replaced with stationary dots stimuli for 800 ms. Subsequently, dots moved in a radial outward pattern (looming motion) on the center of the screen. After 300 ms, the stationary dots were presented for 600 ms. In case of the stationary-dots stimuli, a centered fixation spot was replaced with stationary dots for 1700 ms. Of these trials, the optic flow stimuli occurred 96 times and stationary dots stimuli occurred 264 times.

Visual stimuli were presented on the screen in front of a subject. The subjects were required to maintain centered visual fixation throughout the presentation of all visual stimuli. Two types of stimuli, namely stationary dots and optic flow, were used. The stationary dots stimuli consisted of 1000 white dots that were randomly presented on a black background. The optic flow stimuli consisted of animated sequences of 1000 white dots moving in a radial outward with an average dot speed of 15°/s. The subjects underwent a total of 360 trials (120 trials x 3 sets with intervals of tens of seconds between the sets). Of these trials, the optic flow stimuli occurred 96 times and stationary dots stimuli occurred 264 times. Each trial had a duration time of 2.7 sec in both the types of stimuli. In case of the optic flow stimuli, each trial began with a fixation dot shown for a duration of 1000 ms, followed by stationary dots for 800 ms. Then the dots moved in a radial outward pattern (looming motion) on the center of the screen for 300 ms, and the stationary dots appeared for 600 ms (Fig 2).

In case of the stationary-dots stimuli, each trial began with a fixation dot shown for a duration of 1000 ms, followed by stationary dots for 1700 ms (Fig 2). It has been reported that brain activity evoked by visual motion including optic flow task was easily fluctuated, because of long term adaptation/ habituation or fatigue [27]. To avoid such fluctuation of neural responses to the optic flow stimuli, we used the stationary dots stimuli, and set the optic flow stimuli and the stationary dots stimuli to occur randomly at a ratio of 4:11.

### MEG recordings and MRI scans

The MEG measurements were performed using a MEG system (MEG vision PQA160C; Ricoh Company, Ltd., Kanazawa, Japan). The system consisted of a 160-channel whole-head coaxial gradiometer. The MEG signals were sampled at 1,000 Hz per channel with a 0.1–200 Hz band-pass filter. In a magnetically shielded room, the position of the head within the helmet was determined by magnetic marker coils attached at five locations on the surface of the head as fiduciary points to the landmarks (nasion and pre-auricular points). Using the Sigma Excite HD 1.5T System (GE Yokogawa Medical Systems Ltd., Milwaukee, WI, USA), all subjects underwent T1-weighted MRI studies. For co-registration of MEG with structural MRI images, T1-weighted MRI was performed with spherical lipid markers correspondingly placed at the five MEG fiduciary points. These MRI images consisted of 158 sequential horizontal slices of 1.2 mm thickness, with a resolution of 512 × 512 points in a field of view of 261 × 261 mm.

### Data processing

MEG data of 96 optic flow trials were analyzed for each subject using a MATLAB-based analysis software, FieldTrip. Sensor-level data pre-processing was performed as follows. The data were digitally processed using band-pass (1–30 Hz) filters, and eye-movement and cardiac artifacts were removed by identifying them using an independent component analysis. A stimulus onset in each trial, which was identified with a measured trigger signal, was defined as a start time of the optic flow, and the epochs of interest were set from –300 to 1000 ms. Among them, the epochs with muscle artifacts were removed using an automatic artifact rejection tool implemented in the FieldTrip. The remaining cleaned epochs were averaged in each subject to increase the signal-to-noise ratio of the optic flow response.

Source localizations on individual brains with voxel size of 8 mm were performed for the epoch-averaged signals for each subject by employing the array-gain constraint minimum-norm spatial filter with recursively updated gram matrix (AGMN-RUG) [28], resulting in voxel-level source time courses. The six regions of interests (ROIs), left/right Dorsal Broadmann area 9/46 (A9/46d), Broadmann area 46 (A46), and Ventral Broadmann area 9/46 (A9/46v) that cover the DLPFC, and the eight ROIs, left/right V1, V5/MT+, SPL and IPL that cover the dorsal stream, were set based on the Brainnetome atlas [29]. As event-related fields in the epoch-averaged signals were observed from 100 ms to 300 ms in all the subjects, neural responses of the ROIs to the optic flow were accordingly evaluated by maximum power of source activity within the time interval. The maximum power was determined for each subject by selecting a maximum value among the voxel-level time courses in each ROI. We defined a ROI-level time course as the voxel-level time course with the maximum power.

### Statistical analyses

For discrete variables, we used χ^2^ test. For continuous variables, we checked homoscedasticity of variances using the Levene test. We used the Student’s t-test when the variance was homoscedasticity, and the Welch’s t-test when the variance was heteroscedasticity. We compared the clinical characteristics, MMSE score, WMS-R scores, and the maximum power of each ROIs between the AD-MCI and CU groups. Regarding the maximum power, one-way analysis of variance was utilized to compare for the six ROIs that covered the DLPFC. Multiple comparisons were used with the Turkey method. A correlation between the maximum power of the six ROIs covering the DLPFC and MMSE or WMS-R scores was analyzed for each ROI using Spearman’s correlation test. Accuracies to diagnose AD-MCI for the each six ROIs covering the DLPFC were assessed by the receiver operating characteristic (ROC) analysis. In this study, the optimal cutoff value (OCV) was defined as the cutoff point with the maximum value of the sum of the value of sensitivity and specificity. We further assessed the comparison of the maximum power and the ROC analysis in right-handed participants only. All data were presented as mean (SD), unless otherwise specified. We considered *P* < 0.05 as statistically significant. The ROC analyzes were performed with EZR (Saitama Medical Center, Jichi Medical University, Saitama, Japan), which is a graphical user interface for R (The R Foundation for Statistical Computing, Vienna, Austria). More precisely, it is a modified version of R commander software designed to add statistical functions frequently used in biostatistics [30]. Other statistical analyses were performed using the SPSS software package (version 23; SPSS Inc., Chicago, IL).

## Results

### Subjects’ characteristics

A total of 11 AD-MCI patients and 20 CU subjects participated in this study. The subject characteristics are shown in Table 1. The AD-MCI and CU groups were not significantly different in age, gender, or education period (Table 1). MMSE scores and WMS-R scores except attention/ concentration, were significantly lower in the AD-MCI group than in the CU group (Table 1).

**Table 1:** Clinical characteristics of subjects with CU and AD-MCI.

	CU	AD-MCI	Levene test	*P* value
n (women)	20 (13)	11 (5)	-	0.449
Mean Age, years (SD)	73.5 (5.1)	69.6 (9.9)	0.004	0.252
Mean education period, years (SD)	10.9 (2.6)	10.8 (2.5)	0.999	0.893
Mean MMSE, (SD)	28.4 (1.8)	26.0 (2.6)	0.129	0.006
Mean WMS-R (Verbal memory), (SD)	98.2 (15.7)	74.4 (16.2)	0.697	< 0.001
Mean WMS-R (Visual memory), (SD)	100.3 (17.2)	78.7 (15.2)	0.963	0.002
Mean WMS-R (General memory), (SD)	98.8 (13.8)	73.4 (13.3)	0.747	< 0.001
Mean WMS-R (Attention/ Concentration), (SD)	98.9 (11.5)	91.9 (15.3)	0.067	0.159
Mean WMS-R (Delayed recall), (SD)	96.7 (14.2)	63.3 (10.1)	0.334	< 0.001

Abbreviations: AD-MCI, mild cognitive impairment due to Alzheimer’s disease; CU, cognitively unimpaired participants; MMSE, Mini-Mental State Examination; WMS-R, Wechsler Memory Scale Revised.

There were no patients with visuospatial problems, as assessed by the intersecting pentagon copying test. Of the patients with AD-MCI, five were diagnosed with a high likelihood and six with intermediate likelihood (S1 and S2 Tables). All the MCI patients were converted to AD dementia [31] within 8 years from MEG recording at the MCI stage (S1 and S2 Tables).

### Comparison of the maximum power between the AD-MCI and CU groups

**Fig 3:**
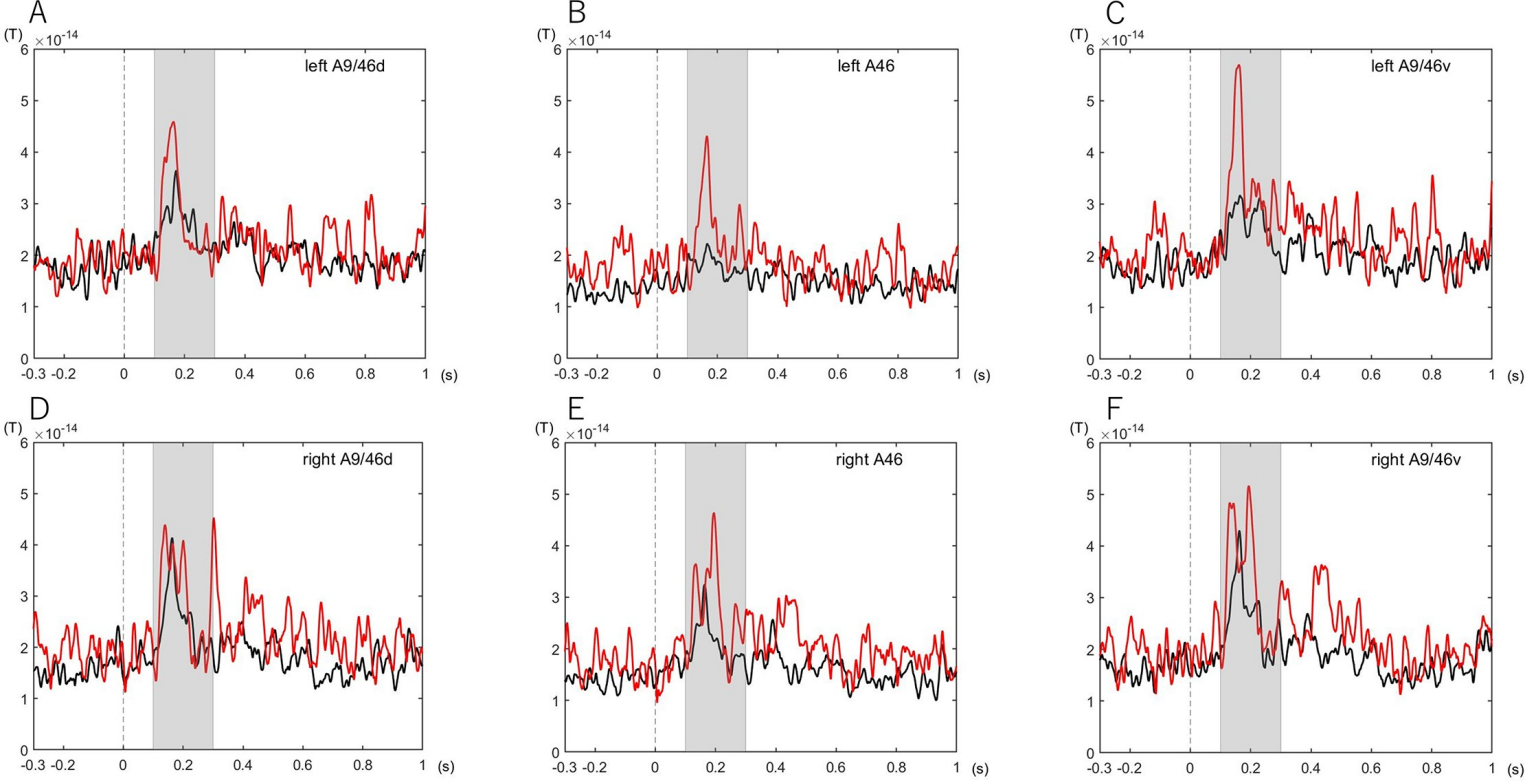
The mean levels of the ROI-level time course in the AD-MCI and CU groups. The ROI-level time courses are showed in the left A9/46d (A), left A46 (B), left A9/46v (C), right A9/46d (D), right A46 (E) and right A9/46v (F). Red line and black line represent the mean levels of the ROI-level time course of AD-MCI and CU groups, respectively. The mean levels of the ROI-level time course in the left A46 (B) and of the left A9/46v (C) are significantly higher in AD-MCI (red line) than in CU (black line) between 100 ms and 300 ms. Abbreviations: A9/46d, Dorsal Broadmann area 9/46; A9/46v, Ventral Broadmann area 9/46; A46, Broadmann area 46; AD-MCI, mild cognitive impairment due to Alzheimer’s disease; CU, cognitively unimpaired participants; ROI, region of interest.

The mean levels of the ROI-level time course of AD-MCI and CU groups in the A9/46d, A46, A9/46v are shown in Fig 3. The mean levels of the ROI-level time course were elevated in patients with AD-MCI than in subjects with CU in left A46 and in left A9/46v (Fig 3B and 3C).

**Fig 4:**
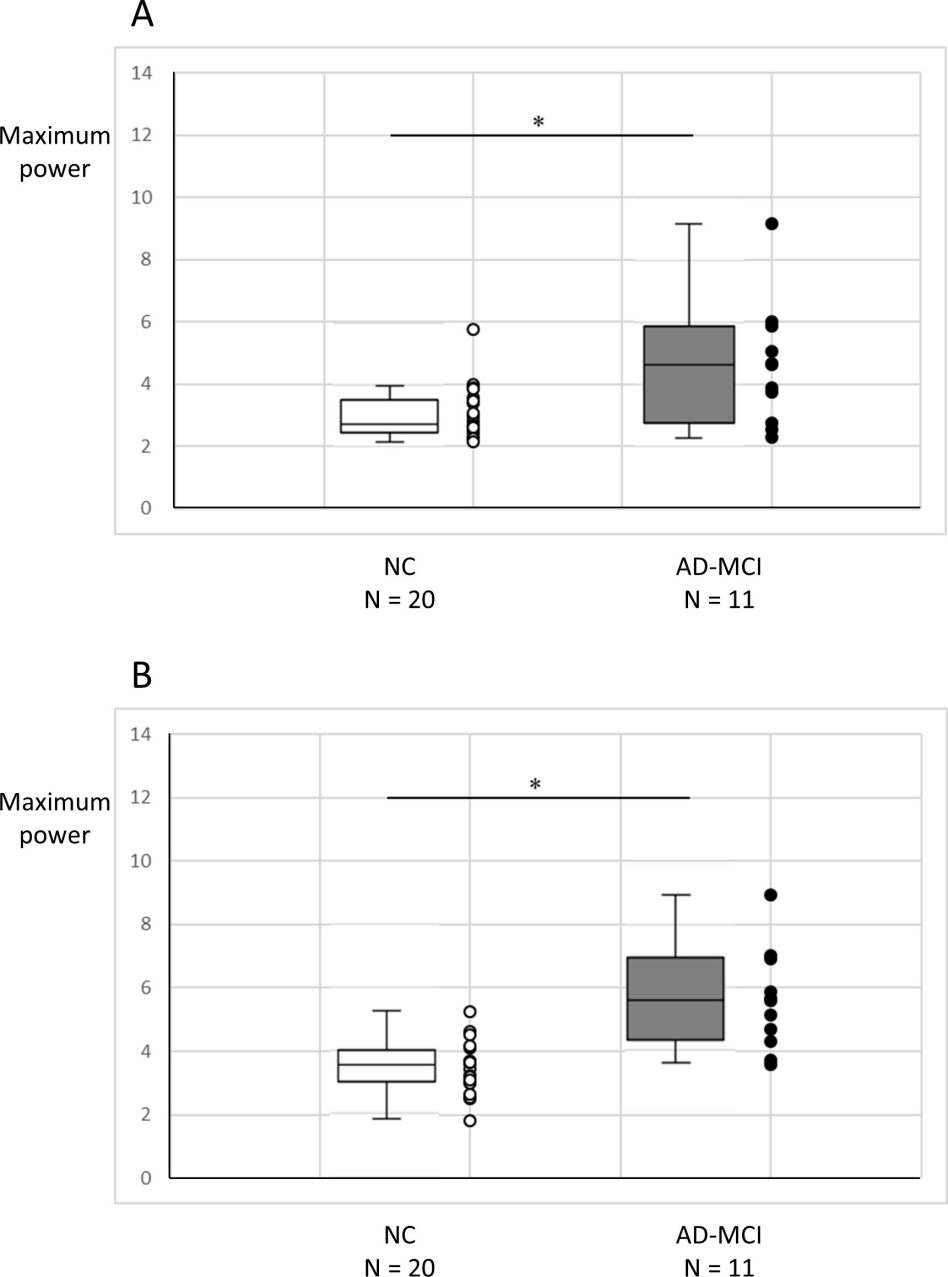
The maximum power distribution in the left A46 (A) and in the left A9/46v (B). The maximum power in the left A46 and in the left A9/46v are significantly higher in AD-MCI than in CU. * *P* < 0.05. Abbreviations: A9/46v, Ventral Broadmann area 9/46; A46, Broadmann area 46; AD-MCI, mild cognitive impairment due to Alzheimer’s disease; CU, cognitively unimpaired participants.

The maximum power in the left A46 and left A9/46v were significantly higher in the AD-MCI group than in the CU group (*P* = 0.029, *P* = 0.001, respectively) during the optic flow task (Fig 4A and 4B).

No significant difference was noted between the AD-MCI and CU groups for any other regions of DLPFC and the dorsal stream (S3 and S4 Tables). In the CU group, the maximum power of the ROIs covering the DLPFC were significantly different (*P* = 0.001); multiple comparisons with the Turkey method showed that the maximum power of the right A9/46d and A9/46v were significantly higher than that of the left A46 (*P* = 0.002, *P* = 0.009, respectively) (S3 Table). On the other hand, there was no significant difference in the AD-MCI group (S3 Table).

**Fig 5:**
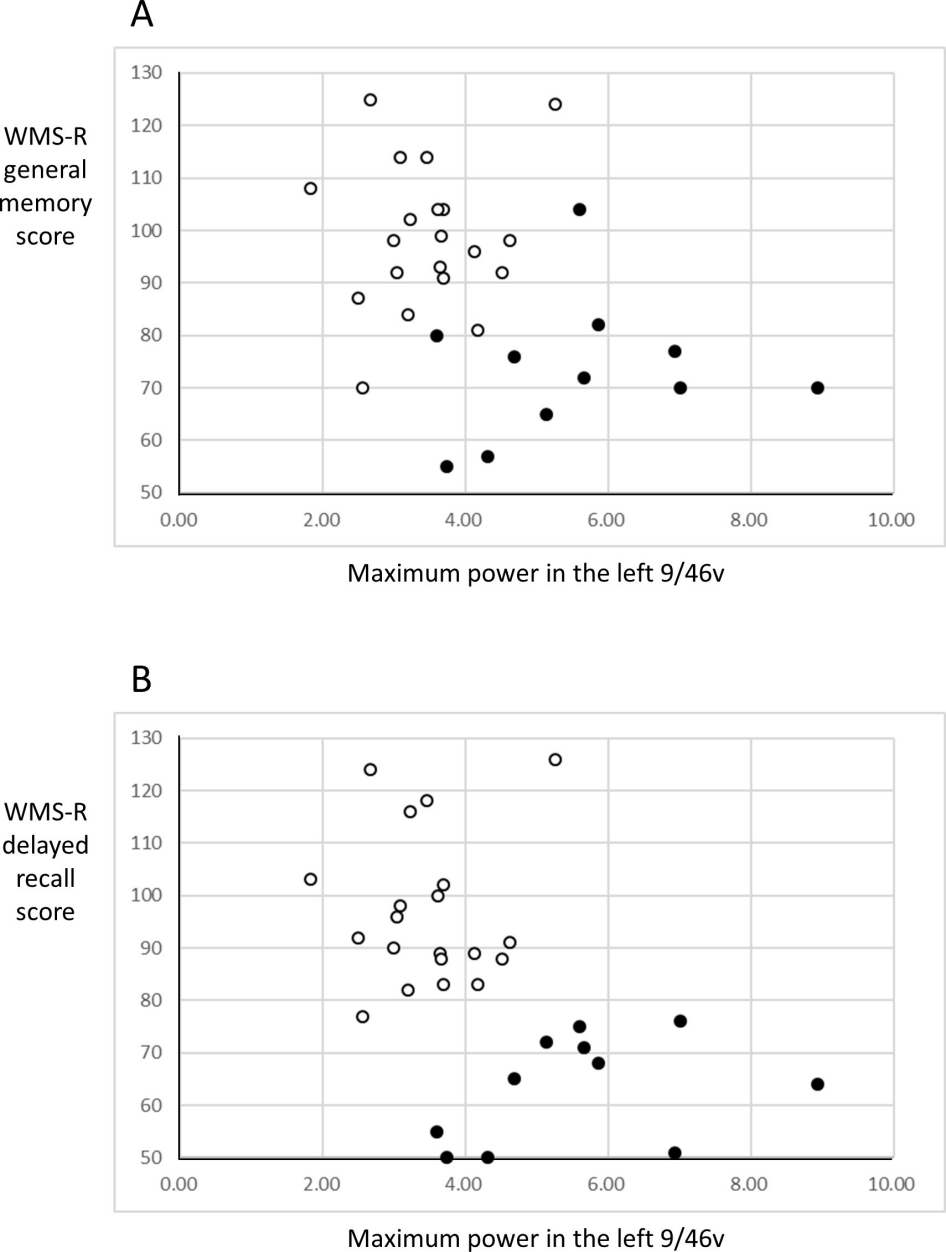
The correlation between the maximum power in the left A9/46v and in the WMS-R general memory score (A) and the WMS-R delayed recall score (B). Open circle represents CU and closed circle represents AD-MCI. Abbreviations: A9/46v, Ventral Broadmann area 9/46; AD-MCI, mild cognitive impairment due to Alzheimer’s disease; CU, cognitively unimpaired participants; WMS-R, Wechsler Memory Scale Revised.

The maximum power in the left A9/46v significantly correlated with the WMS-R general memory score and delayed recall score (*r* = −0.414, *P* = 0.021 and *r* = −0.554, *P* = 0.001, respectively) (Fig 5A and 5B).

Regarding neural responses in right and left DLPFC, the maximum power of the right A9/46d and A9/46v showed significantly higher than that of the left A46 (*P* = 0.002, *P* = 0.009, respectively) in the CU group as described above (S3 Table). When we limited to the right-handed participants, the significance of higher maximum power in the left A9/46v of AD-MCI during the optic flow task remained (*P* = 0.001), whereas the significance in the left A46 disappeared. In addition, the correlation between the maximum power in the left A9/46v and the WMS-R delayed recall score remained significant (*r* = −0.493, *P* = 0.008) in the right-handed participants; however, the significant correlation with the WMS-R general memory score disappeared.

### Discrimination accuracy between the AD-MCI and CU groups

**Fig 6:**
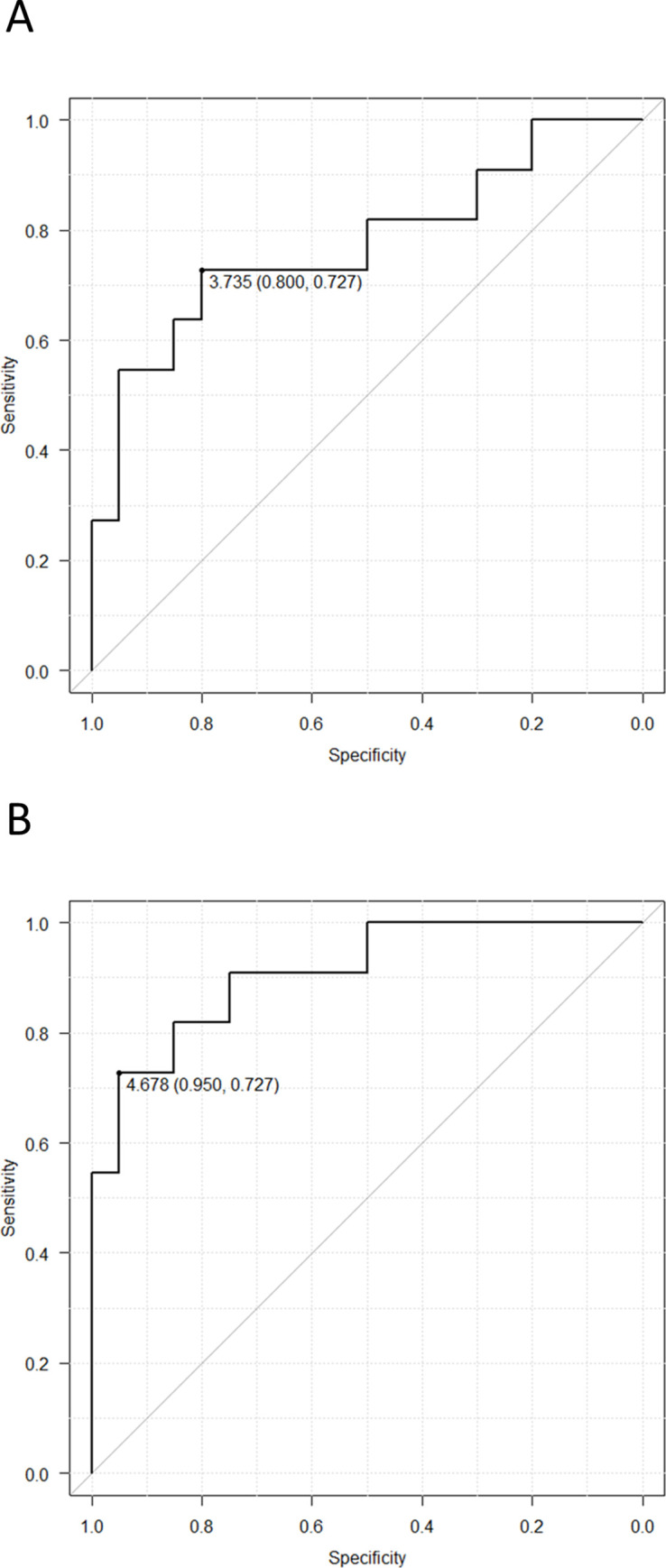
The ROC curves of the maximum power in the left A46 (A) and in the left A9/46v (B) to distinguish AD-MCI from CU. Abbreviations: A9/46v, Ventral Broadmann area 9/46; A46, Broadmann area 46; AD-MCI, mild cognitive impairment due to Alzheimer’s disease; CU, cognitively unimpaired participants; ROC, receiver-operating characteristic.

For distinguishing AD-MCI from CU, the sensitivities/specificities at the OCV were 0.727/ 0.800 and 0.727/ 0.950, and the area under the curves in ROC analyses were 0.77 and 0.90 for the left A46 and left A9/46v, respectively (Fig 6A and 6B). When we limited in the right-handed participants, the area under the curves were 0.71 and 0.88 for the left A46 and left A9/46v, respectively.

## Discussion

We demonstrated that the AD-MCI group showed significantly higher maximum power than that of the CU group in the left DLPFC during the optic flow task. In addition, the maximum power in the left DLPFC was negatively correlated with higher cognitive function. Our results indicated that cognitive decline that reflects Alzheimer pathology progression at the MCI stage would be associated with a change in the processing of optic flow stimulations in the left DLPFC.

### Increased activity of DLPFC in AD-MCI

The DLPFC has been considered as the key component of attention [15] and decision-making function [17]. It has been reported that the visuospatial attention signals were vulnerable in neurodegenerative diseases, including AD, because of cholinergic disruption [15]. When we make decisions related to visual motion, such as optic flow, the lateral intra-parietal area (LIP) was reported to work for the accumulation of visual evidence [32] and DLPFC for the integration of visual information [33]. We supposed that cholinergic vulnerability of the visuospatial attention signals of AD-MCI group might affect increased activity of the DLPFC as the compensation strategy. Less activity has been reported in the PPC, which includes LIP in patients with amnestic MCI when compared to cognitively unimpaired group during the memory retrieval task in a fMRI study [34], suggesting the metabolic disruption of PPC in AD-MCI. It has also been reported that increased activation in DLPFC and decreased activation in posterior areas are observed during the memory task [35], and this DLPFC activation has been thought to be due to the compensation mechanisms [35]. Although no significant differences were observed in the neural responses to optic flow stimuli in the PPC between AD-MCI and CU groups, the increased activity of the DLPFC was negatively correlated with higher cognitive function in our study. Therefore, we supposed that function of the posterior areas including PPC of AD-MCI group might be deteriorated and the DLPFC was activated due to the compensation mechanisms in our study. In summary, the increased activity of the DLPFC might be the compensation strategy for cholinergic vulnerability of the visuospatial attention signals and the deteriorated function of the PPC.

### Laterality of increased brain activities in DLPFC

We observed the increased brain activities in the left DLPFC, and not in the right DLPFC. It is unknown whether the laterality of this neural pathway is affected by the dominant hand. The results remained almost unchanged when we limited to the right-handed subjects. It has been reported that left and right DLPFCs are associated with verbal and non-verbal information, respectively [36]. Moreover, the fMRI study suggested that the optic flow task activates the right hemisphere including DLPFC but not the left DLPFC [11]; the subjects of these studies [11, 36] were young adults. The Hemispheric Asymmetry Reduction in Older adults (HAROLD) model [37] explained changes in brain activation with age-associated neurocognitive decline. Regarding the lateralization of DLPFC, right DLPFC activation was specific to young adults [38], while, in older adults, the activation was not only in the right DLPFC but also in the left DLPFC [38]. Our analyses revealed that the neural responses to optic flow stimuli were significantly higher in the right DLPFC than that in the left DLPFC in CU group, but not in AD-MCI group. This suggests that neural responses to optic flow stimuli in the right DLPFC are enough to perception in CU group, and neural responses in the left DLPFC might be unnecessary to be activated. On the other hand, in AD-MCI group, neural responses in the left DLPFC might be necessary to perceive optic flow stimuli and to keep visuospatial attention because of cholinergic dysfunction of brain network for attention signals in neurodegenerative conditions [15]. In other words, differences in the brain activity between the AD-MCI and CU groups during the optic flow stimuli with looming motion might be associated with the compensation strategy of AD-MCI to achieve an equal level of performance as CU. When neurodegeneration is advanced to the frontal lobe including DLPFC in cases such as severe AD dementia, neural responses of both sides of DLPFC are speculated to be lower than those in CU group. Further studies are hence necessary to clarify the brain networks of optic flow perception, including laterality of DLPFC in cognitively normal elderly and subjects with advanced AD dementia and other neurodegenerative disorders.

### MEG as a tool for evaluating visuospatial functions to detect AD-MCI

Differences in the brain activity between the AD-MCI and CU groups might be associated with visuospatial impairment. However, none of the subjects had visuospatial problems clinically. Therefore, MEG measurements during the optic flow stimuli with looming motion might be able to detect the abnormalities about visuospatial function early even at the subclinical stage. Increasing age is the most significant risk factor for AD [39]. It is necessary to develop quick and easy methods for diagnosing AD-MCI that can be applied to many people who have reached a certain age. We demonstrated that the MEG activities in the left DLPFC during optic flow task, which took only 15 min, displayed certain diagnostic accuracies to detect AD-MCI. The measurement of MEG during the optic flow task could be an effective diagnostic procedure for AD-MCI, requiring minimum effort from elderly persons.

### Limitation

The present study had several limitations. First, the number of subjects was small. Second, we did not obtain pathological verification of the AD diagnosis for any patient. Third, further studies with patients with MCI due to various types of dementia, such as MCI with Lewy bodies are necessary to conclude whether the MEG findings in this study are specific for AD-MCI.

## Conclusions

In conclusion, our results suggest that AD-MCI would be associated with alteration of the brain function of the left DLPFC during the perception of optic flow stimuli with looming motion and that MEG analysis of the brain activity in the left DLPFC in the optic flow would be useful in discriminating between AD-MCI and CU.

## Supporting information

S1 Table The cognitive tests data of patients with mild cognitive impairment due to Alzheimer’s disease group. (DOCX)

S2 Table The data of biomarker measurement of patients with mild cognitive impairment due to Alzheimer’s disease group. (DOCX)

S3 Table The mean maximum power in each ROIs. (DOCX)

S4 Table The mean maximum power in each ROIs that covered the dorsal stream. (DOCX)
