# Supplementary material for: MEG activity of the dorsolateral prefrontal cortex during optic flow stimulations detects mild cognitive impairment due to Alzheimer’s disease
Source: PLoS One. 2021 Nov 5;16(11):e0259677. doi: 10.1371/journal.pone.0259677 (PMC8570504; doi:10.1371/journal.pone.0259677)
Supplement: S1 Table — (DOCX) [file pone.0259677.s001.docx]

**Supplementary table 1. The cognitive tests data of patients with mild cognitive impairment due to Alzheimer’s disease group.**

| **No** | **Age** | **Sex** | **MMSE** | **WMS-R**  **Verbal memory** | **Visual memory** | **General memory** | **Attention/ Concentration** | **Delayed recall** |
| --- | --- | --- | --- | --- | --- | --- | --- | --- |
| 1 | 53 | M | 30 | 55 | 75 | 55 | 105 | <50 |
| 2 | 56 | M | 28 | 92 | 64 | 80 | 101 | 55 |
| 3 | 60 | F | 27 | 60 | 70 | 57 | 102 | <50 |
| 4 | 65 | F | 25 | 67 | 100 | 77 | 114 | 51 |
| 5 | 68 | M | 23 | 111 | 90 | 104 | 85 | 75 |
| 6 | 74 | F | 28 | 74 | 68 | 70 | 79 | 64 |
| 7 | 74 | M | 29 | 65 | 91 | 72 | 112 | 71 |
| 8 | 75 | M | 23 | 75 | 98 | 82 | 84 | 68 |
| 9 | 78 | F | 26 | 75 | 98 | 82 | 84 | 68 |
| 10 | 81 | F | 22 | 73 | 57 | 65 | 69 | 72 |
| 11 | 82 | M | 25 | 85 | 64 | 76 | 76 | 65 |

Abbreviations: MMSE, Mini-Mental State Examination; WMS-R, Wechsler memory scale-revised index score.
