# Supplementary material for: MEG activity of the dorsolateral prefrontal cortex during optic flow stimulations detects mild cognitive impairment due to Alzheimer’s disease
Source: PLoS One. 2021 Nov 5;16(11):e0259677. doi: 10.1371/journal.pone.0259677 (PMC8570504; doi:10.1371/journal.pone.0259677)
Supplement: S2 Table — (DOCX) [file pone.0259677.s002.docx]

**Supplementary table 2. The data of biomarker measurement of patients with mild cognitive impairment due to Alzheimer’s disease group.**

|  |  |  | **Biomarkers of Aβ** | | **Biomarker of tau** | **Biomarkers of neurodegeneration** | | **Duration from MEG recording at MCI stage to conversion to dementia (months)** |
| --- | --- | --- | --- | --- | --- | --- | --- | --- |
| **No** | **Age** | **Sex** | **CSF Aβ_42_ (pg/mL)** | **Amyloid PET** | **CSF phosphorylated tau (pg/mL)** | **FDG-PET** | **MTA score** |  |
| 1 | 53 | M | 633 | Positive | 118 | Positive | 2 | 22 |
| 2 | 56 | M | 255 | Positive | 47 | Positive | 2 | 31 |
| 3 | 60 | F | NT | Positive | NT | Positive | 1 | 25 |
| 4 | 65 | F | 760 | Positive | 131 | Positive | 1 | 6 |
| 5 | 68 | M | 368 | NT | 83 | NT | 3 | 31 |
| 6 | 74 | F | NT | NT | NT | NT | 2 | 60 |
| 7 | 74 | M | NT | NT | NT | NT | 2 | 72 |
| 8 | 75 | M | NT | NT | NT | NT | 2 | 36 |
| 9 | 78 | F | NT | NT | NT | NT | 2 | 84 |
| 10 | 81 | F | NT | NT | NT | NT | 3 | 36 |
| 11 | 82 | M | NT | NT | NT | NT | 3 | 96 |

Cutoff values of CSF Aβ_42_ and CSF phosphorylated tau for separation of normal were 490 pg/mL and 49 pg/mL, respectively. Amyloid PET positive means cortical amyloid deposition such as unilateral binding in one or more cortical brain region, that to be positive for Alzheimer pathology. FDG-PET positive means reduction in the metabolism in the temporoparietal lobe, precuneus and/or posterior cingulate gyrus, to be positive for neurodegeneration pathology. MTA ≥ 2 was considered pathological atrophy.

Abbreviations: Aβ, amyloid beta protein; CSF, cerebrospinal fluid; MCI, mild cognitive impairment; MEG, magnetoencephalography; MRI, magnetic resonance imaging; MTA, medial temporal atrophy; NT, not tested; PET, positron-emission tomography; ptau, phosphorylated tau protein.
