# Supplementary material for: MEG activity of the dorsolateral prefrontal cortex during optic flow stimulations detects mild cognitive impairment due to Alzheimer’s disease
Source: PLoS One. 2021 Nov 5;16(11):e0259677. doi: 10.1371/journal.pone.0259677 (PMC8570504; doi:10.1371/journal.pone.0259677)
Supplement: S3 Table — (DOCX) [file pone.0259677.s003.docx]

**Supplementary table 3. The Mean maximum power in each ROIs.**

|  | **CU** | **AD-MCI** | ***P* value**  CU vs. AD-MCI |
| --- | --- | --- | --- |
| R A9/46d, Mean (SD) | 4.52 (1.32)* | 5.46 (1.42) | 0.078 |
| L A9/46d, Mean (SD) | 4.08 (1.17) | 4.78 (2.21) | 0.344 |
| R A46, Mean (SD) | 3.59 (1.30) | 4.48 (1.81) | 0.151 |
| L A46, Mean (SD) | 3.05 (0.86) | 4.59 (1.96) | 0.029 |
| R A9/46v, Mean (SD) | 4.35 (1.44)* | 5.20 (1.71) | 0.179 |
| L A9/46v, Mean (SD) | 3.47 (0.81) | 5.58 (1.58) | 0.001 |

* *P* < 0.05 versus L A46 in CU group

Abbreviations: A9/46d, Dorsal Broadmann area 9/46; A9/46v, Ventral Broadmann area 9/46; A46, Broadmann area 46; AD-MCI, mild cognitive impairment due to Alzheimer’s disease; CU, cognitively unimpaired; L, left; R, right; ROI, regions of interest.
