# Supplementary material for: MEG activity of the dorsolateral prefrontal cortex during optic flow stimulations detects mild cognitive impairment due to Alzheimer’s disease
Source: PLoS One. 2021 Nov 5;16(11):e0259677. doi: 10.1371/journal.pone.0259677 (PMC8570504; doi:10.1371/journal.pone.0259677)
Supplement: S4 Table — (DOCX) [file pone.0259677.s004.docx]

**Supplementary table 4. The Mean maximum power in each ROIs that covered the dorsal stream.**

|  | **CU** | **AD-MCI** | ***P* value**  CU vs. AD-MCI |
| --- | --- | --- | --- |
| R V1, Mean (SD) | 6.23 (3.48) | 5.14 (2.17) | 0.354 |
| L V1, Mean (SD) | 6.71 (2.70) | 5.70 (2.65) | 0.327 |
| R V5/MT, Mean (SD) | 15.11 (10.4) | 14.45 (10.75) | 0.868 |
| L V5/MT, Mean (SD) | 16.07 (7.87) | 14.57 (9.46) | 0.640 |
| R SPL, Mean (SD) | 10.31 (3.42) | 9.58 (3.89) | 0.588 |
| L SPL, Mean (SD) | 7.79 (4.05) | 9.66 (5.79) | 0.300 |
| R IPL, Mean (SD) | 10.52 (4.42) | 9.35 (6.76) | 0.565 |
| L IPL, Mean (SD) | 9.12 (4.76) | 10.23 (6.29) | 0.585 |

Abbreviations: AD-MCI, mild cognitive impairment due to Alzheimer’s disease; CU, cognitively unimpaired; IPL, inferior parietal lobule; L, left; R, right; ROI, regions of interest; SPL, superior parietal lobule; V1, visual cortex 1; V5/MT, visual cortex5/ middle temporal
